# Gastric-Type Expression Signature in Hepatocellular Carcinoma

**DOI:** 10.3390/ijms25126588

**Published:** 2024-06-15

**Authors:** Rita Szodorai, Laura Banias, Ilona Kovalszky, Katalin Dezső, Zsolt Kovács, Simona Gurzu

**Affiliations:** 1 Department of Pathology, George Emil Palade University of Medicine, Pharmacy, Science and Technology, 540139 Targu Mures, Romania; ritaszodorai@gmail.com (R.S.); laurabanias@gmail.com (L.B.); 2 Department of Pathology, Clinical County Emergency Hospital Targu Mures, 540140 Targu Mures, Romania; kovacska_zsoltkovacs@yahoo.com; 3 Department of Pathology and Experimental Cancer Research, Faculty of Medicine, Semmelweis University, 1085 Budapest, Hungary; kovalszky.ilona@med.semmelweis-univ.hu (I.K.); dezso.katalin@semmelweis.hu (K.D.); 4 Research Center of Oncopathology and Translational Research (CCOMT), 540139 Targu Mures, Romania; 5 Romanian Academy of Medical Sciences, 030167 Bucharest, Romania

**Keywords:** hepatocellular carcinoma, epithelial–mesenchymal transition, VSIG1, TTF-1, vimentin, gastric type

## Abstract

It is known that V-set and immunoglobulin domain containing 1 (VSIG1) is a cell–cell adhesion molecule that can serve as an indicator of better survival in patients with gastric cancer. Its interaction with cytoplasmic thyroid transcription factor 1 (TTF-1) has been hypothesized to characterize gastric-type HCC, but its clinical importance is far from understood. As VSIG1 has also been supposed to be involved in the epithelial–mesenchymal transition (EMT) phenomenon, we checked for the first time in the literature the supposed interaction between VSIG1, TTF-1, and Vimentin (VIM) in HCCs. Immunohistochemical (IHC) stains were performed on 217 paraffin-embedded tissue samples that included tumor cells and normal hepatocytes, which served as positive internal controls. VSIG1 positivity was seen in 113 cases (52.07%). In 71 out of 217 HCCs (32.71%), simultaneous positivity for VSIG1 and TTF-1 was seen, being more specific for G1/G2 carcinomas with a trabecular architecture and a longer OS (*p* = 0.004). A negative association with VIM was revealed (*p* < 0.0001). Scirrhous-type HCC proved negative for all three examined markers. The present paper validates the hypothesis of the existence of a gastric-type HCC, which shows a glandular-like architecture and is characterized by double positivity for VSIG1 and TTF-1, vimentin negativity, and a significant OS.

## 1. Introduction

Hepatocellular carcinoma (HCC) exerts a significant global burden in terms of cancer-related mortality. Presently, HCC holds the fifth position among the most prevalent malignancies worldwide and assumes the role of the second leading cause of cancer-related death in the male population, trailing behind only lung cancer [1,2,3].

A high percentage of HCC cases, ranging from 80% to 90%, arise in individuals diagnosed with cirrhosis. The annual incidence of HCC in cirrhotic patients is 2–4%. It is the predominant primary liver tumor, constituting over 90% of all primary hepatic neoplasms. Liver cancer continues to pose a substantial worldwide health challenge, with an anticipated incidence surpassing one million cases by the year 2025 [3,4].

Although significant improvements have been made in the therapeutic management of HCC, its survival rate is still low. To better modulate its therapy, it is important to understand hepatocarcinogenesis. 

In a recently published study, we postulated that, although human V-set and immunoglobulin domain containing 1 (VSIG1) is known as a gastric-related biomarker, its unusual cytoplasmic positivity can be seen in a gastric-type HCC that shows double positivity for VSIG1 and thyroid transcription factor 1 (TTF-1) [1]. In this paper, we aimed to explore the interaction between these two biomarkers in a larger cohort of HCC cases that did not include the cases from our previous study [1]. As VSIG1 is also supposed to be involved in the epithelial-to-mesenchymal transition (EMT) phenomenon, vimentin (VIM) was added to complete the immune-profile of these cancer cells. No data about this interaction have been published yet.

VSIG1 is known as an adhesion molecule that is highly expressed in the epithelium of the gastric mucosa [5,6,7]. Its significant loss at both the mRNA and protein levels within the membrane of gastric tumor cells, compared to non-cancerous gastric mucosa, is used as a negative prognostic factor, indicating its potential role as a tumor suppressor gene [6,7,8]. No data about the possible prognostic or predictive role of VSIG1 in HCC are known. 

TTF-1 belongs to the NKx2 homeobox protein family and is expressed in the nuclei of the normal epithelial cells of the thyroid and lung, but also in thyroid and pulmonary carcinomas [9]. In HCC, Wieczorek et al. noted that TTF-1 stains the cytoplasm of tumor cells in HCC and a small percentage of metastatic liver carcinomas [10,11]. However, the cytoplasmic staining of TTF-1 is not specific for HCC, because it can also be present in cholangiocarcinoma [9].

This study aimed to explore the interaction between VSIG1, TTF-1, and VIM as a potential carcinogenic pathway of the gastric-type HCC in a representative cohort of cases, and to check the prognostic impact of this new histological subtype [1,12].

## 2. Results

### 2.1. Clinicopathological Parameters

In this study, a cohort of 217 patients diagnosed with HCC was included. Males proved to predominate (69.58%) compared with females (30.41%), with a male–female ratio of 3:1. The age of the patients ranged from 31 to 91 years. Half of the diagnosed tumors were multifocal and additionally associated with hepatitis B or C, steatosis, and alcohol-related cirrhosis. More than half of the cases (*n* = 127, 57.60%) were categorized as high-grade HCC. Although in the 8th edition of AJCC, HCC is staged as 1A, 1B, 2, 3A, 3B, 4A, and 4B, for statistical purposes, we used the combined stages as 1, 2, and 3 (Table 1). No cases in the fourth stage were included.

### 2.2. VSIG1 Correlation with Clinicopathological Parameters

The cytoplasmic expression of VSIG1 was detected in 113 cases. Strong positivity (3+) was identified in 36 cases, moderate positivity (2+) in 54 cases, and mild positivity (1+) in the other 23 cases. The expression profile of VSIG1 demonstrated a downregulation in pT3 stages compared to pT1/2 stages, but the differences were significant only if they were interpretated in correlation with histological dedifferentiation. In G4 solid tumors, VSIG1 tended to be negative, especially in the solid-schirous histological subtype. This feature persisted in both unifocal and multicentric tumors, with a conspicuous prevalence in pT3-staged-HCC, in contrast to pT1-staged-HCC, which were mostly G1 tumors with a trabecular architecture (Table 2).

### 2.3. TTF-1 Correlation with Clinicopathological Parameters

TTF-1 proved to be overexpressed in well- and moderately differentiated (G1/G2) carcinomas with a trabecular architecture, more so than in poorly differentiated cases with a solid-schirous architecture (Table 3).

### 2.4. Vimentin Correlation with Clinicopathological Parameters

Vimentin expression was detected in fewer than 17% of cases, and tended to be more overexpressed in HCC with a solid-scirrhous architecture (Table 4).

### 2.5. Correlation between VSIG1, TTF-1, VIM, and Clinicopathological Parameters

**Figure 1:**
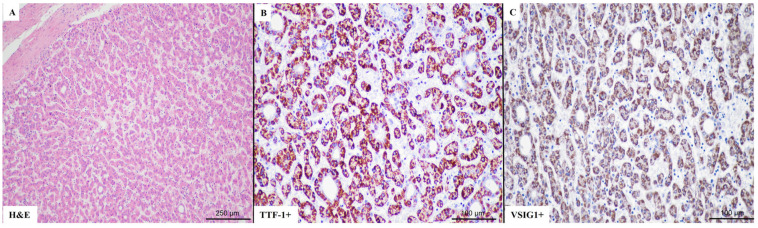
A representative case of well-differentiated hepatocellular carcinoma (**A**–**C**), with double positivity for TTF-1 (**B**) and VSIG1 (**C**).

**Figure 2:**
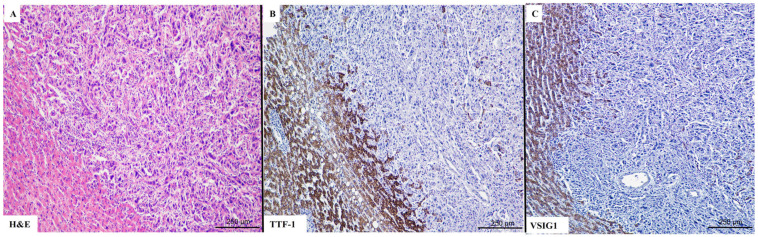
A representative case of poorly differentiated hepatocellular carcinoma (**A**–**C**), with double negative stain for TTF-1 (**B**) and VSIG1 (**C**).

A direct correlation was seen between VSIG1 and TTF-1 positivity (Table 5). VIM tended to be lost in VSIG1- and/or TTF-1-positive cases. VSIG1+/TTF-1+ cases (Figure 1, which are known as “gastric-type HCCs” [1], proved to be well-differentiated HCC with a trabecular architecture, whereas double negativity (Figure 2) was more specific for schirous-type HCC (Table 5).

**Figure 3:**
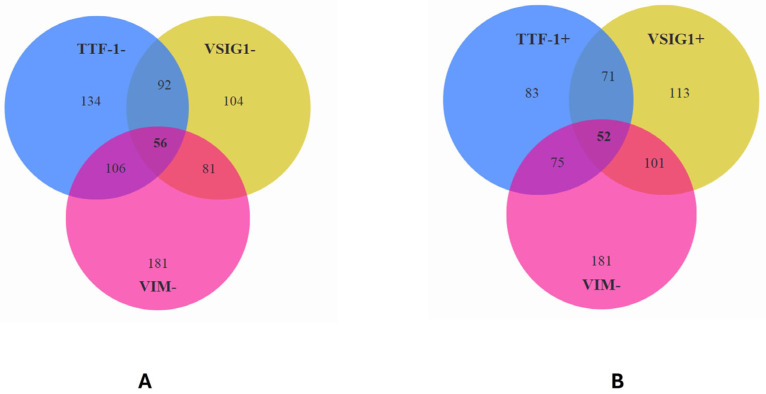
Interaction between VSIG1, TTF-1, and vimentin in hepatocellular carcinoma proves a direct correlation between TTF-1 and VSIG1, especially for positive cases. (**A**) Most cases with double positivity or double negativity for TTF-1 and VSIG1 did not express vimentin (**A**,**B**).

From a cohort of 83 cases positive for TTF-1, 113 cases positive for VSIG1, and 181 cases negative for VIM, we identified 52 cases expressing a gastric-type HCC profile which were negative for VIM (TTF-1+ VSIG1+ VIM−) (Figure 3).

When triple negativity for VSIG1, TTF-1, and VIM was checked, 56 cases demonstrated a triple-negative HCC phenotype (Figure 3). This was predominantly seen in poorly differentiated HCCs. All the HCCs with a scirrhous histologic type presented triple negativity for the above-checked markers (Table 6).

Only three cases showed triple positivity for VSIG1, TTF-1, and VIM, all of them being poorly differentiated trabecular HCCs.

### 2.6. Survival Rate

**Figure 4:**
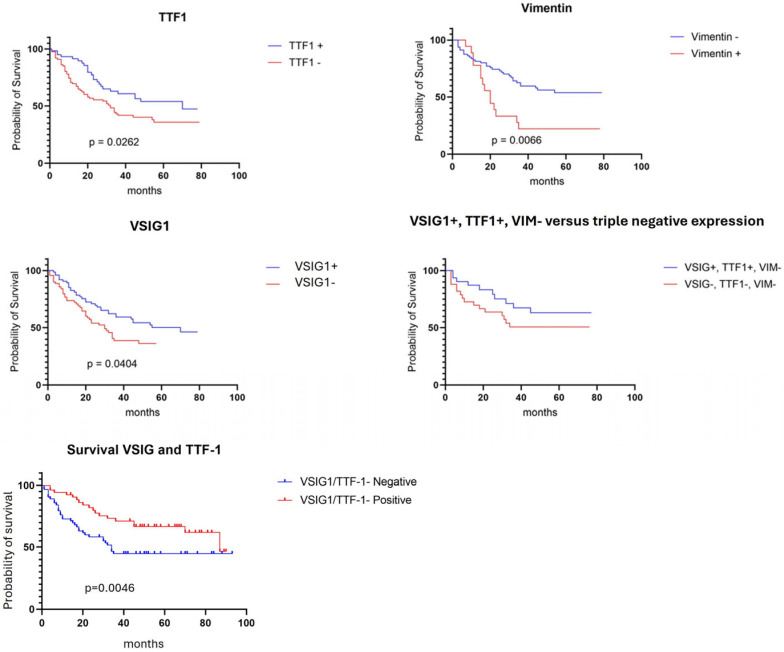
In patients with hepatocellular carcinoma, VSIG1, TTF-1, and vimentin can serve as independent prognostic parameters. Double positivity for VSIG1 and TTF-1 reflects a longer OS, independently of vimentin expression.

The Kaplan–Meier curves revealed a significant correlation between OS and the expression of all three IHC markers. A longer OS was proved for patients with HCC that showed positivity for VSIG1 and/or TTF-1, respectively, with an absence of vimentin expression. Although triple-negative HCCs tended to show a lower OS rate, the difference was not significant. Double positivity for VSIG1 and TTF-1 was a stronger indicator of a longer OS than the individual expression of the two markers (Figure 4).

## 3. Discussion

The VSIG1 protein was firstly described in 2006 as a gastric-mucosa-specific cell adhesion molecule [13] with an important role in cancer cell biology for cell-to-cell interaction, migration, proliferation, and invasion. Scanlan et al. [13] described that VSIG1 is a junction molecule (JAM) that belongs to the JAM family, and is predominantly expressed in normal gastric mucosa and the testis, but is also expressed in gastric adenocarcinoma, esophageal, ovarian, and colon cancers [14].

In gastric cancer, the membrane to cytoplasmic translocation of VSIG1 or its loss in the invasion front are considered to be an indicator of unfavorable prognosis. No data about the subcellular expression of VSIG1 in HCC are known yet [15]. In a previous study, we showed that VSIG1 cytoplasmic positivity is seen in normal hepatocytes, but can be infrequently seen in HCC. Membrane positivity is not a characteristic stain for hepatocytes or HCC [1]. Based on an embryological theory, a common origin was supposed between hepatoid-type gastric carcinoma and a subtype of HCC. Due to these reasons, VSIG1-positive HCCs were called “gastric-type HCCs” [1].

In the present study, in a larger cohort of HCCs, the previous theory was validated in a completely new and representative cohort of cases. As in other carcinomas, in HCC, loss of the VSIG-1-mediated cell–cell adhesion seems to be associated with a worse prognosis, but the mechanisms of the tumor suppressor function of VSIG1 are still not understood [1,14].

In hepatoid carcinomas of the stomach and gastric-type HCC, the cytoplasmic expression of TTF-1 can also be seen. For the first time in the literature, TTF-1/VSIG1 co-expression in HCC proved to be a valuable positive prognostic indicator, being especially identified in G1/G2 trabecular HCCs. Its prognostic impact needs to be, however, correlated with tumor stage.

As VSIG1 has also been supposed to act in correlation with epithelial–mesenchymal transition (EMT) genes, such as E-cadherin, N-cadherin, and VIM [1,12], this interaction was also checked. EMT is a complex biological process, entailing the transformation of polarized, immotile epithelial cells into migratory mesenchymal cells [16]. In many carcinomas, EMT is accompanied by vimentin positivity [17,18,19]. Given the involvement of the cytoplasmic domain of VSIG1 in the assembly of tight junctions [13], we hypothesized that the loss of VSIG1 might promotie EMT. Recent findings underscore vimentin’s crucial role as a mediator of EMT-related effects induced by osteopontin (OPN), a multifunctional protein implicated in cancer progression. The inhibition of vimentin significantly attenuates OPN-induced alterations in EMT marker expression, providing compelling evidence for vimentin involvement in mediating the effects of OPN on EMT processes [20]. The overexpression of VIM may play an important role in the metastatic potential of HCC [20]. Although the exact role of vimentin is far from being understood in HCC, its positivity in tumor cells remains a negative prognostic indicator. Gastric-type HCC does not express vimentin and it seems that its behavior is not dictated by the EMT phenomenon.

When the independent prognostic value of the three markers was checked, both VSIG1 and TTF-1 expression correlated with an improved OS compared to negative tumors. Gastric-type HCC, characterized by simultaneous positivity for TTF-1 and VSIG1 positivity, showed a better OS than TTF-1 only or VSIG1 only positive HCCs. Conversely, an elevated expression of VIM in HCC is associated with decreased OS rates, independent of TTF-1 or VSIG1 expression.

These observations suggest that TTF-1 and VSIG1 may serve as favorable prognostic markers, while VIM expression may indicate a poorer clinical outcome in HCC patients. The immunohistochemical profile of gastric-type HCC characterized by VSIG1+ TTF-1+ VIM− [1] exhibits a potentially favorable prognosis compared to triple-negative HCC.

## 4. Materials and Methods

### 4.1. Selection of HCC Cases

We performed a retrospective observational analysis of clinicopathological data extracted from a consecutive cohort of patients diagnosed with HCC. The data were collected between 2013 and 2022, drawing from clinical records maintained at the Semmelweis University Hospital in Budapest, Hungary, and the Emergency Clinical County Hospital of Târgu Mureș, Romania. As we intended to validate the data from one of our previous studies [1], to avoid overlapping, the cases that were included in the present study are different from those of the other cohort. They were diagnosed between 2013 and 2022, while those from our first study were managed between 2011 and 2014 [1]. 

This study involves cases of HCC in which patient inclusion was contingent upon the requirement of a minimum three-months follow-up following surgical intervention. Patients who had undergone antecedent oncological therapy before surgery were excluded from the study. The study was granted approval by the Ethical Committee of the Emergency Clinical County Hospital of Târgu Mureș, Romania, no 11780/20.05.2021 and Semmelweis University Regional and Institutional Committee of Science and Research Ethics, no 155/2021.

The creation of individual patient profiles was accomplished through the amalgamation of pertinent patient characteristics, including age, gender, tumor classification, histological grading, and tumor stage, upon the 8th edition of the American Joint Committee on Cancer (AJCC) Cancer Staging Manual [1,2]. All cases were histologically assessed based on the 5th edition of the World Health Organization (WHO) Manual for Digestive System Tumors [12].

### 4.2. Immunohistochemistry 

In our study, immunohistochemistry (IHC) staining was performed on formalin-fixed paraffin-embedded tissue (FFPE) blocks comprising hepatocellular carcinoma (HCC) cells and adjacent non-tumoral tissues. The IHC staining was performed using the automated IHC staining system Dako AutostainerLink 48 (Dako Agilent Technologies, santa Clara, United States).

Thin sections measuring 3–5 µm in thickness underwent deparaffinization and rehydration, followed by the inhibition of endogenous peroxidase activity using Dako EnVisionTM FLEX Peroxidase-Blocking Reagent, with an incubation period of 5 min at room temperature. Antigen retrieval was performed at a high temperature for 30–40 min using a high-pH retrieval solution, succeeded by a 20 min incubation at room temperature with Dako EnVisionTM FLEX/HRP detection reagent. EnVisionTM FLEX diaminobenzidine was employed for stain development, with Mayer’s hematoxylin used for nuclei counterstaining. 

The IHC panel included antibodies against VSIG1 (rabbit polyclonal HPA036311, Sigma-Aldrich; diluted at 1:200), TTF-1 (clone 8G7G3/1, DAKO; diluted at 1:50), and VIM (clone V9, DAKO; diluted at 1:600). Incubation was performed at room temperature for 45 min. Following the incubation period, diaminobenzidine (DAB) solution was used for immunostaining development, followed by counterstaining with Mayer’s hematoxylin. Normal hepatocytes served as an internal positive control for cytoplasmic VSIG1 and TTF-1.

### 4.3. Interpretation of IHC Stains

The examination and interpretation of the IHC staining on the slides were conducted with a Nikon Eclipse E400 laboratory microscope. Digital image acquisition was facilitated by employing the high-definition 5-megapixel Nikon Digital Sight DS-U3 microscope camera controller with the Nikon NIS-Elements universal software platform, version 3. 

**Figure 5:**
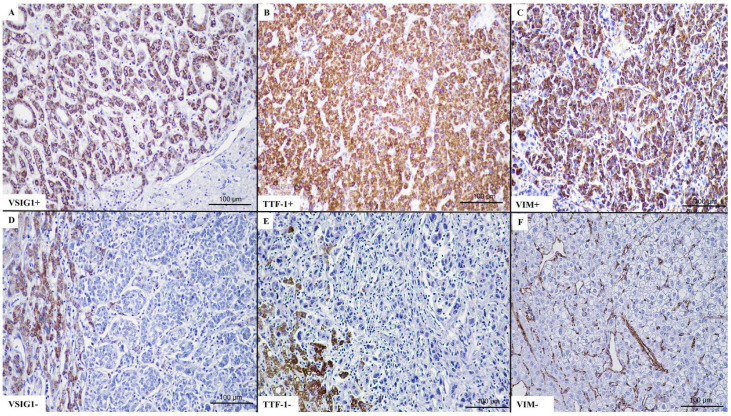
Conventional evaluation of VSIG1 positivity (**A**–**C**). Positive cytoplasmatic staining for (**A**) VSIG1, (**B**) TTF-1, and (**C**) VIM, with lack of expression in representative cases (**D**–**F**). In cases with no positivity for (**D**) VSIG-1 and (**E**) TTF-1, the adjacent non-tumoral hepatocytes served as internal positive control. For vimentin, vascular structures were used as internal positive control (**F**).

The evaluation of the cytoplasmic expressions of VSIG1, TTF-1, and VIM (Figure 5) was performed with a predefined threshold set at 5%. For VSIG1, based on the IHC stain intensity and percentage of positive tumor cells, cases were classified into four categories: negative (0), weakly positive (1+), moderate positivity (2+), and strongly positive (3+) [1]. For statistical purposes, the negative and positive cases were considered.

The IHC stains were blinded and independently evaluated by two senior pathologists (SG and IJ) and one junior pathologist (RS). When the results of any interpretation were divergent, the case was re-discussed by the team. 

**Figure 6:**
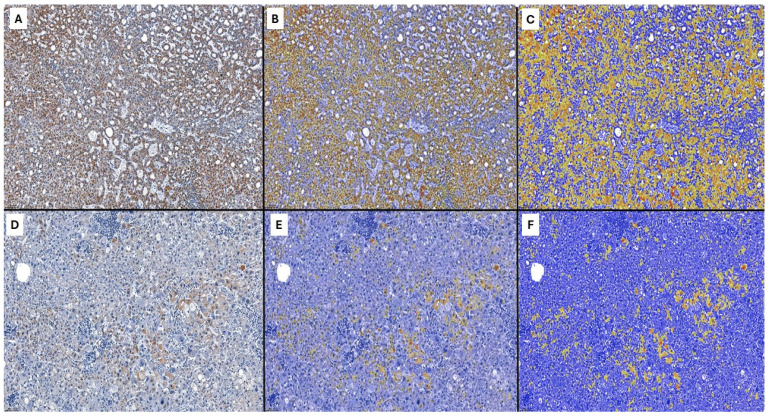
Automatic evaluation of VSIG1 positivity with the QuantCenter software, version 3.0 (**A**–**C**). Representative images of positive (76.50%) cytoplasmatic expression of VSIG1 versus (**D**–**F**), a negative case (1.48%). The yellow-brownish areas represent the positive hepatocytes. Caption: ob. × 20, 0.100 mm.

For the reliability of the interpretation, the slides were also scanned using the PANNORAMIC 250 Flash III slide scanner (3DHISTECH Ltd.—Budapest, Hungary). An analysis of the cytoplasmic expression of VSIG-1 was also performed with the multiple-module image analysis QuantCenter software, version 3.0 (https://www.3dhistech.com/research/software/latest-releases/ accessed on 1 May 2024), designed by The Digital Pathology Company—3DHISTECH (Figure 6).

### 4.4. Statistical Analysis

The enrolled cases underwent statistical evaluation through the GraphPad Prism software (version 8). The examination of the correlation between clinicopathological characteristics and IHC markers employed multivariate analysis techniques, including the Pearson χ^2^, chi-square, and Fisher’s exact tests. Statistical significance was defined as *p* < 0.05. Overall survival (OS) rates were estimated using the same software, complemented by Kaplan–Meier curves. The median follow-up period post-surgery was 34 months (range 3 to 93 months). 

## 5. Conclusions

Emphasizing the expression of VSIG1 and TTF-1 may serve as a favorable prognostic factor, indicating improved OS rates, while the overexpression of vimentin indicates a poorer prognosis.

## Figures and Tables

**Table 1:** Clinicopathological and immunohistochemical parameters of patients with HCC.

Parameters	N = 217
Age (years), median (range)	65.48 (31–91 years)
Gender (male/female)	151/66
Tumor architecture (uni-/multifocal)	93/124
Tumor size—the diameter of the larger tumor (mm)	43.89 (7–140)
pT stage—8th AJCC edition (1/2/3)	97/89/31
Histologic type (trabecular/solid-acinar/solid-clear cells/solid-scirrhous)	90/47/56/24
Grade of differentiation (G1/G2/G3/G4)	13/79/81/44
Vascular invasion (present/absent)	95/122
Liver cirrhosis (present/absent)	143/74
Hepatitis (yes/no)	105/112
VSIG1 (0/1+/2+/3+)	104/23/54/36
Vimentin (negative/positive)	181/36
TTF-1 (negative/positive)	134/83

**Table 2:** Correlation of clinicopathological and immunohistochemical parameters with VSIG1 expression in hepatocellular carcinoma.

Parameter	VSIG1 (*n* = 217)		*p* Value
	Negative (n = 104, 47.92%)	Positive (n = 113, 52.07%)	
Age (yrs.), median (range)	65.14 (31–82 years)	64.07 (39–91 years)	0.86
Gender			0.91
Male (n = 151)	72 (69.23%)	79 (69.91%)	
Female (n = 66)	32 (30.76%)	34 (30.08%)	
Tumor architecture			0.49
Unifocal (n = 93)	42 (40.38%)	51 (45.13%)	
Multicentric (n = 124)	62 (59.61%)	62 (54.86%)	
pT stage—8th AJCC edition			0.26
pT1 (n = 98)	49 (47.11%)	49 (43.36%)	
pT2 (n = 88)	37 (35.57%)	51 (45.13%)	
pT3 (n = 31)	18 (17.30%)	13 (11.50%)	
Histologic type			<0.0001
Trabecular (n = 90)	33 (31.73%)	57 (50.44%)	
Solid-acinar (n = 47)	24 (23.07%)	23 (20.35%)	
Solid-scirrhous (n = 24)	21 (20.19%)	3 (2.65%)	
Solid-clear cells (n = 56)	26 (25%)	30 (26.54%)	
Grade of differentiation			<0.0001
G1 + G2 (n = 92)	23 (22.11%)	69 (61.06%)	
G3 + G4 (n = 125)	81 (77.88%)	44 (38.93%)	
Vascular invasion			0.49
Present (n = 96)	49 (47.11%)	47 (41.59%)	
Absent (n = 121)	55 (52.88%)	66 (58.40%)	
Liver cirrhosis			0.88
Present (n = 143)	68 (65.38%)	75 (66.37%)	
Absent (n = 74)	36 (34.61%)	38 (33.62%)	
Hepatitis B or C history			0.68
Yes (n = 98)	45 (43.26%)	53 (46.90%)	
No (n = 119)	59 (56.73%)	60 (53.09%)	
Vimentin—tumor cells			0.02
Negative (n = 182)	81 (77.88%)	101 (89.38%)	
Positive (n = 35)	23 (22.11%)	12 (10.61%)	
TTF-1—tumor cells			<0.0001
Negative (*n* = 134)	92 (88.46%)	42 (37.16%)	
Positive (*n* = 83)	12 (11.53%)	71 (62.83%)	

**Table 3:** Correlation of clinicopathological and immunohistochemical parameters with TTF-1 expression in HCC.

Parameter	TTF-1 (*n* = 217)		*p* Value
	Negative (n = 134, 61.75%)	Positive (n = 83, 38.24%)	
Age (yrs.), median (range)	64.54 (31–91 years)	64.39 (39–81)	0.84
Gender			0.87
Male (n = 151)	94 (70.14%)	57 (68.67%)	
Female (n = 66)	40 (29.85%)	26 (31.32%)	
Tumor architecture			0.40
Unifocal (n = 108)	70 (52.23%)	38 (45.78%)	
Multicentric (n = 109)	64 (47.76%)	45 (54.21%)	
pT stage—8th AJCC edition			0.26
pT1 (n = 96)	59 (44.02%)	37 (44.57%)	
pT2 (n = 90)	52 (38.80%)	38 (45.78%)	
pT3 (n = 31)	23 (17.16%)	8 (9.63%)	
Histologic type			0.07
Trabecular (n = 90)	51 (38.05%)	39 (46.98%)	
solid-acinar (n = 47)	30 (22.38%)	17 (20.48%)	
solid-clear cells (n = 56)	33 (24.62%)	23 (27.71%)	
solid-scirrhous (n = 24)	20 (14.92%)	4 (4.81%)	
Grade of differentiation			<0.0001
G1 + G2 (n = 92)	43 (32.08%)	49 (59.03%)	
G3 + G4 (n = 125)	91 (67.91%)	34 (40.96%)	
Vascular invasion			0.88
present (n = 96)	60 (44.77%)	36 (43.37%)	
absent (n = 121)	74 (55.22%)	47 (56.62%)	
Liver cirrhosis			0.76
Present (n = 142)	89 (66.41%)	53 (63.85%)	
Absent (n = 75)	45 (33.58%)	30 (36.14%)	
Hepatitis B or C history			0.26
yes (n = 105)	69 (51.49%)	36 (43.37%)	
no (n = 112)	65 (48.50%)	47 (56.62%)	
Vimentin—tumor cells			0.03
negative (n = 181)	106 (79.10%)	75 (90.36%)	
positive (n = 36)	28 (20.89%)	8 (9.63%)	
VSIG1—tumor cells			<0.0001
negative (n = 134)	92 (68.65%)	12 (14.45%)	
positive (n = 83)	42 (31.34%)	71 (85.54%)	
0/1+/2+/3+	(92/13/21/8)	(12/10/33/28)	

**Table 4:** Correlation of clinicopathological and immunohistochemical parameters with VIM expression in HCC.

Parameter	VIM (*n* = 217)		*p* Value
	Negative (n = 181, 83.41%)	Positive (n = 36, 16.58%)	
Age (yrs.), median (range)	65.37 (39–84)	67.04 (59–91)	0.54
Gender			0.87
Male (n = 149)	125 (69.06%)	24 (66.66%)	
Female (n = 68)	56 (30.93%)	12 (33.33%)	
Tumor architecture			0.18
Unifocal (n = 127)	110 (60.77%)	17 (47.22%)	
Multicentric (n = 90)	71 (39.22%)	19 (52.77%)	
pT stage—8th AJCC edition			0.35
pT1 (n = 96)	84 (46.40%)	12 (33.33%)	
pT2 (n = 95)	76 (41.98%)	19 (52.77%)	
pT3 (n = 26)	21 (11.60%)	5 (13.88%)	
Histologic type			0.05
Trabecular (n = 92)	80 (44.19%)	12 (33.33%)	
Solid-acinar (n = 35)	30 (16.57%)	5 (13.88%)	
Solid-clear cells (n = 72)	60 (33.14%)	12 (33.33%)	
Solid-scirrhous (n = 18)	11 (6.07%)	7 (19.44%)	
Grade of differentiation			0.46
G1 + G2 (n = 94)	76 (41.98%)	18 (50%)	
G3 + G4 (n = 123)	105 (58.01%)	18 (50%)	
Vascular invasion			0.34
Present (n = 96)	84 (46.40%)	23 (63.88%)	
Absent (n = 121)	97 (53.59%)	13 (36.11%)	
Liver cirrhosis			0.08
Present (n = 133)	116 (64.08%)	17 (47.22%)	
Absent (n = 84)	65 (35.91%)	19 (52.77%)	
Hepatitis B or C history			0.73
Yes (n = 93)	79 (43.64%)	14 (38.88%)	
No (n = 112)	102 (56.35%)	22 (61.11%)	
TTF-1—tumor cells			0.04
Negative (n = 134)	106 (58.56%)	28 (77.77%)	
Positive (n = 83)	75 (41.43%)	8 (22.22%)	
VSIG1—tumor cells			0.03
Negative (*n* = 105)	81 (44.75%)	24 (66.66%)	
Positive (*n* = 112)	100 (55.24%)	12 (33.33%)	

**Table 5:** Correlation of clinicopathological parameters and double VSIG1/TTF-1 immuno-profile of HCC.

Parameters	VSIG1+ TTF-1+ = 71	VSIG1− TTF-1− = 92	*p* Value
Age (yrs.), median (range)	64 (39–81 years)	65.5 (31–91 years)	0.87
Gender			0.47
Male (n = 112)	49 (69.01%)	63 (68.47%)	
Female (n = 51)	22 (30.98%)	29 (31.52%)	
Tumor architecture			0.32
Unifocal (n = 78)	32 (45.07%)	46 (50%)	
Multicentric (n = 85)	39 (54.92%)	46 (50%)	
pT stage—8th AJCC edition			0.38
pT1 (n = 75)	32 (45.07%)	43 (46.73%)	
pT2 (n = 66)	32 (45.07%)	34 (36.95%)	
pT3 (n = 22)	7 (9.85%)	15 (16.30%)	
Histologic type			0.006
Trabecular (n = 64)	35 (49.29%)	29 (31.52%)	
Solid-acinar (n = 35)	14 (19.71%)	21 (22.82%)	
Solid-clear cells (n = 41)	19 (26.76%)	22 (23.91%)	
Solid-scirrhous (n = 23)	3 (4.22%)	20 (21.73%)	
Grade of differentiation			<0.0001
G1 + G2 (n = 62)	44 (61.97%)	18 (19.56%)	
G3 + G4 (n = 101)	27 (38.02%)	74 (80.43%)	
Vascular invasion			0.34
Present (n = 73)	30 (42.25%)	43 (46.73%)	
Absent (n = 100)	41 (57.74%)	49 (53.26%)	
Liver cirrhosis			0.52
Present (n = 104)	45 (63.38%)	59 (64.13%)	
Absent (n = 59)	26 (36.61%)	33 (35.86%)	
Hepatitis			0.18
Yes (n = 81)	32 (45.07%)	49 (53.26%)	
No (n = 82)	39 (54.92%)	43 (46.73%)	

**Table 6:** Correlation of clinicopathological parameters with triple VSIG1/TTF-1/VIM immno-profile of HCC.

Parameters	VSIG1+ TTF-1+ VIM− = 52	VSIG1− TTF-1− VIM− = 56	*p* Value
Age (yrs.), median (range)	65.20 (39–91)	64.8 (30–82)	0.87
Gender			0.40
Male	34 (65.38%)	41 (73.21%)	
Female	18 (34.61%)	15 (26.78%)	
Tumor architecture			0.43
Unifocal	28 (53.85%)	35 (62.5%)	
Multicentric	24 (46.15%)	21 (37.5%)	
pT stage—8th AJCC edition			0.75
pT1 (n = 49)	23 (44.23%)	26 (46.42%)	
pT2 (n = 46)	23 (44.23%)	23 (41.07%)	
pT3 (n = 13)	6 (11.53%)	7 (12.05%)	
Histologic type			0.02
Trabecular (n = 47)	27 (51.92%)	20 (35.71%)	
Solid-acinar (n = 18)	7 (13.46%)	11 (19.64%)	
Solid-clear cells (n = 35)	18 (34.61%)	17 (30.35%)	
Solid-scirrhous (n = 8)	0 (0.00%)	8 (14.28%)	
Grade of differentiation			<0.0001
G1 + G2 (n = 43)	31 (59.61%)	12 (21.42%)	
G3 + G4 (n = 65)	21 (40.38%)	44 (78.57%)	
Vascular invasion			0.70
Present	24 (46.15%)	28 (50%)	
Absent	28 (53.85%)	28 (50%)	
Liver cirrhosis			0.32
Present	28 (53.85%)	36 (64.28%)	
Absent	24 (46.15%)	20 (35.71%)	
Hepatitis			0.33
Yes	20 (38.46%)	27 (48.21%)	
No	32 (61.53%)	29 (51.78%)	

## Data Availability

All data are available in the authors own database.
